# Development of a Rapid and Simple LC-MS/MS Method for the Quantitation of Baricitinib in Human Plasma and Aqueous Humor

**DOI:** 10.21627/kqcara81

**Published:** 2026-06-01

**Authors:** Xiping Li, Xian Zhang, Chengliang Zhang, Hengyi Yu, Yu Liu, Xufang Sun

**Affiliations:** Department of Pharmacy, Tongji Hospital, Tongji Medical College, Huazhong University of Science and Technology, 1095 Jiefang Avenue, Wuhan 430030, China; Department of Ophthalmology, Tongji Hospital, Tongji Medical College, Huazhong University of Science and Technology, 1095 Jiefang Avenue, Wuhan 430030, China; Department of Pharmacy, Tongji Hospital, Tongji Medical College, Huazhong University of Science and Technology, 1095 Jiefang Avenue, Wuhan 430030, China; Department of Pharmacy, Tongji Hospital, Tongji Medical College, Huazhong University of Science and Technology, 1095 Jiefang Avenue, Wuhan 430030, China; Department of Pharmacy, Tongji Hospital, Tongji Medical College, Huazhong University of Science and Technology, 1095 Jiefang Avenue, Wuhan 430030, China; Department of Ophthalmology, Tongji Hospital, Tongji Medical College, Huazhong University of Science and Technology, 1095 Jiefang Avenue, Wuhan 430030, China

**Keywords:** Noninfectious uveitis, baricitinib, LC-MS/MS, aqueous humor, therapeutic drug monitoring

## Abstract

**BACKGROUND:**

Increasing evidence supports the off-label use of Janus kinase inhibitors for the treatment of noninfectious uveitis. Although several bioanalytical methods for determining baricitinib concentrations have been reported, no methods are currently available for monitoring concentrations at or near noninfectious uveitis lesions. This article aims to develop a rapid, sensitive, and reliable LC-MS/MS method for determining baricitinib concentrations in both plasma and aqueous humor, facilitating future clinical pharmacology research and improving therapeutic management.

**METHODS:**

After single-step protein precipitation and centrifugation, the samples were subjected to direct analysis. Optimal chromatographic performance was achieved using an Ultimate XB-C_18_ column with a mobile phase of 10 mM of ammonium formate and a mixture of methanol, acetonitrile, and isopropanol (0.2% formic acid).

**RESULTS:**

The authors validated the method for the limits of detection and quantification, linearity, accuracy, precision, selectivity, matrix effects, stability, and recovery, with all results meeting guidelines. This method has been successfully applied to monitor baricitinib concentrations in adult patients with noninfectious uveitis, revealing substantial variability in plasma concentrations and unsynchronized pharmacokinetic changes between plasma and aqueous humor.

**CONCLUSION:**

This robust method can be a valuable tool for future clinical pharmacology research and improving therapeutic management.

## Non-Specialist Summary

This study presents a fast, simple lab test to measure the disease-modifying antirheumatic drug baricitinib in both the plasma and aqueous humor. Using small sample volumes and one-step preparation, the LC-MS/MS method is sensitive, accurate, and stable across a wide range of concentrations. Applied to adults with noninfectious uveitis, it revealed large variability in plasma concentrations and a weak correlation between plasma and aqueous humor pharmacokinetics. These findings suggest that therapeutic drug monitoring of baricitinib may be warranted to promote individualized treatment.

## 1. Introduction

Noninfectious uveitis (NIU) is a heterogeneous class of uveitis primarily caused by excessive activation of the autoimmune system, leading to inflammatory lesions in ocular tissues [1]. Damage due to NIU not only involves the eye without a specific cause but can also occur concurrently with various immune-mediated inflammatory diseases (IMIDs), such as psoriasis, Behçet’s disease, sarcoidosis, and Crohn’s disease [2,3]. The standard treatment strategy for NIU primarily relies on the local or systemic administration of corticosteroids and various immunosuppressive agents [4–8]. Long-term use of these medications, however, can lead to severe ocular complications and other systemic adverse drug reactions [9,10]. Moreover, a proportion of patients with NIU, particularly those with more severe disease, do not achieve satisfactory improvement with any of the currently available therapies [11]. Therefore, a significant unmet medical need remains for a more effective and well-tolerated NIU treatment.

The Janus kinase (JAK) superfamily, which includes JAK1, JAK2, JAK3, and tyrosine kinase 2, plays a crucial role in the regulation of multiple cytokine-related inflammatory pathways [12]. JAK inhibitors can effectively suppress inflammation by inhibiting the JAK signal transducer and activator of transcription (JAK-STAT) signaling pathway. Given the close relationship of the pathogenesis of NIU with several autoimmune diseases, as well as the significant role of the JAK-STAT signaling pathway in inflammation and the beneficial prospects of JAK inhibitors in rheumatic diseases, it is speculated that JAK inhibitors could achieve good or even superior therapeutic outcomes in the treatment of refractory NIU [13,14].

Baricitinib is a selective and reversible JAK 1/2 inhibitor that has been approved for the treatment of several IMIDs, but its use in NIU is still off-label. Baricitinib is a highly soluble drug with poor permeability characterized by low or inconsistent bioavailability in different disease populations. The interindividual variability in baricitinib pharmacokinetics (PKs) was 17% to 26% in healthy volunteers and 41% in patients with rheumatoid arthritis [15]. In recent years, increasing evidence has supported its potential in the treatment of concomitant uveitis in many IMIDs [16–18]. However, little information is available regarding the PK profiles of baricitinib in these patients. Notably, the European Medicines Agency (EMA) has highlighted the safety risks of all JAK inhibitors in terms of major cardiovascular problems, malignancy, venous thromboembolism, serious infections, and death [19]. These findings collectively indicate that implementing clinical pharmacology studies and therapeutic drug monitoring (TDM) to balance the cost-benefit and risks of baricitinib is warranted.

The detection conditions and validation results of the quantification method are prerequisites for PK studies. Several publications have described liquid chromatographic–tandem mass spectrometry (LC-MS/MS) methods to determine baricitinib concentrations in human or animal plasma [20–25]. Clinical baricitinib quantification in the aqueous humor of patients with NIU remains unavailable, however. Furthermore, whether the linear or TDM window of baricitinib based on plasma can be extended to the aqueous humor is unclear. This article aims to develop a straightforward, rapid, sensitive, reproducible, and reliable method using LC-MS/MS to quantify baricitinib in both plasma and aqueous humor. This method will promote future clinical studies and the rational use of baricitinib in the treatment of NIU.

## 2. Materials and Methods

### 2.1. Chemical Substances and Laboratory Materials

Reference baricitinib (lot 22Z102-M1; purity, 98.68%) and internal standard (IS) baricitinib-d5 (lot 22T083-L1; purity, 98%) (**Figure 1**) were procured from Shanghai Zzbio Co. Ltd. (Shanghai, China). High-performance liquid chromatography–grade methanol, acetonitrile, dimethyl sulfoxide (DMSO), and ammonium acetate and American Chemical Society–grade formic acid were obtained from Aladdin Co. (Shanghai, China). The ultrapure water used in this study was produced using an ELGA Purelab Flex 3 water system (High Wycombe, United Kingdom). Blank plasma and whole blood from healthy volunteers were donated by the Phase I Clinical Laboratory of Tongji Hospital (ethical approval number: TJ-IRB20220737). Blank aqueous humor was provided by patients who underwent cataract surgery in our hospital. Written informed consent was obtained from all the patients.

**Figure 1.**
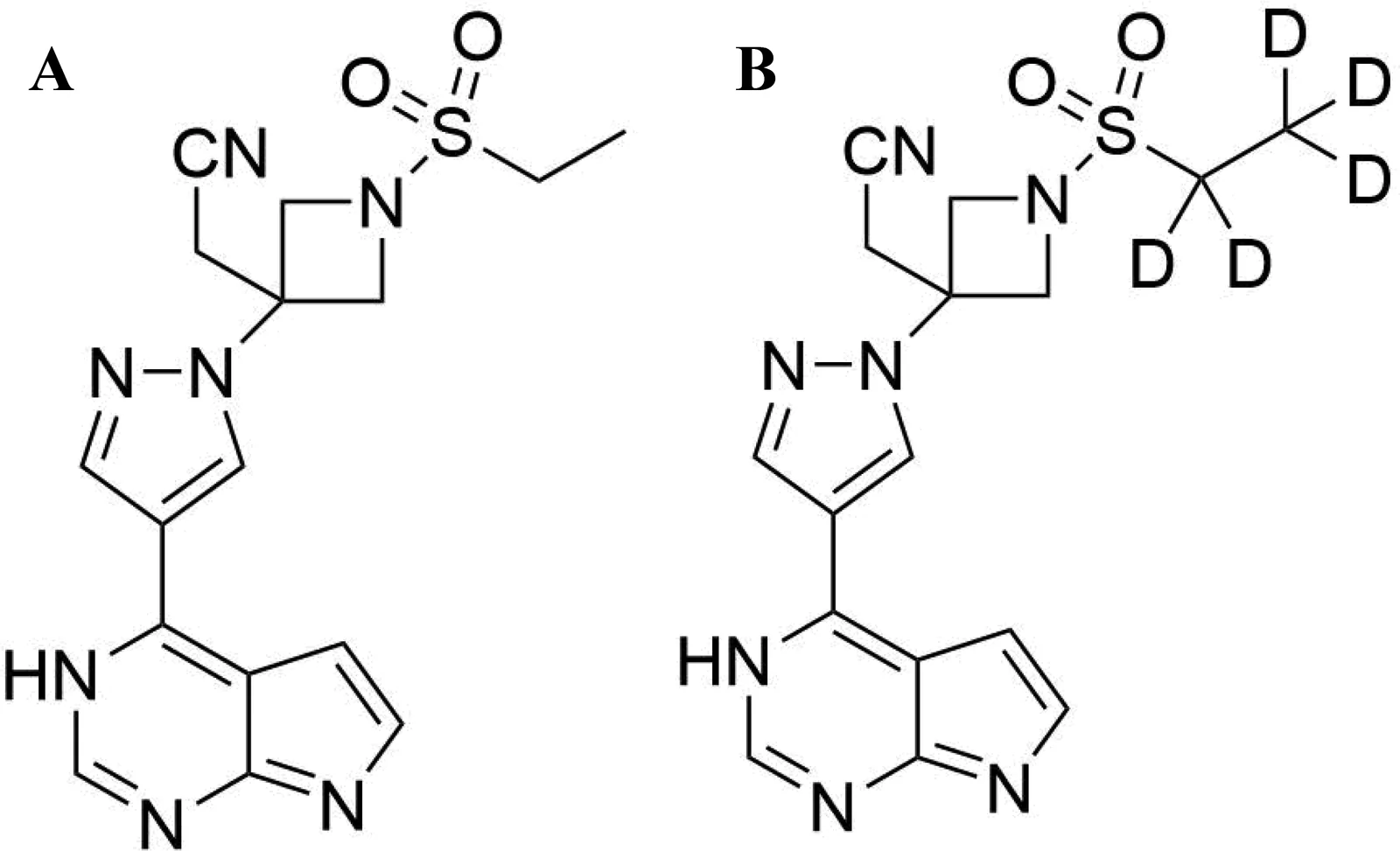
Chemical structure of baricitinib (**A**) and its isotopic IS (**B**).

### 2.2. Preparation of Calibration Standards and Quality Controls

Accurately weighed amounts of baricitinib and baricitinib-d5 (IS) were dissolved in a ratio of methanol to DMSO (40:60, volume/volume percentage [v/v]) to yield standard stock solutions of 1.0 mg/mL and 40 μg/mL, respectively. The baricitinib stock solution was diluted with acetonitrile to obtain a series of standard (4–4,000 ng/mL) and quality control (QC) working solutions (4, 12, 150, and 3,000 ng/mL). Similarly, a 40 ng/mL IS solution was prepared by diluting the stock IS solution.

Calibration standards for baricitinib in human plasma or aqueous humor were prepared at concentrations of 0.2, 0.5, 2, 5, 10, 40, 100, and 200 ng/mL. This was achieved by mixing 20 μL of standard working solutions with 180 μL of blank plasma or aqueous humor. QC samples were independently prepared using the same method. These QC samples included concentrations at 0.20 ng/mL (lower limit of quantification [LLOQ]), 0.6 ng/mL (low-quality control [LQC]), 7.5 ng/mL (medium-quality control [MQC]), and 150 ng/mL (high-quality control [HQC]). Furthermore, a dilution quality control (DQC) sample was prepared at a concentration of 400 ng/mL. Subsequently, the DQC sample was diluted with blank plasma or aqueous humor (dilution factor, 5) to produce an 80 ng/mL dilution test sample.

### 2.3. Sample Preparation

An aliquot of 20 μL of IS working solution (40 ng/mL) was added to a sample of 50 μL of plasma or aqueous humor. The mixture was vortex mixed for 10 s. Subsequently, an aliquot of 100 μL of acetonitrile was added to facilitate protein precipitation. The mixture was then thoroughly extracted on a vortex mixer for 5 min and centrifuged at 12,000 rpm for 10 min at 4 °C. Next, 100 μL of the protein-free supernatant was carefully transferred to a new 1.5 mL plastic vial, followed by 100 μL of an acetonitrile-water solution (50:50, v/v). Finally, a 6 μL sample of the resulting mixture was injected for detection and analysis.

### 2.4. Instrument Settings

LC-MS/MS analysis was performed using a Shimadzu Corp. LC-20A liquid chromatographic system (Kyoto, Japan) coupled to a QTRAP 5500 mass spectrometer (AB Sciex, MA). The mass spectrometer was equipped with an electrospray ionization (ESI) source. Positive multiple reaction monitoring (MRM) mode was used to analyze the samples. The quantitative and confirming MRM mass transitions (m/z) were 372.3 *>* 186.1 and 372.2 > 251.1 for baricitinib and 377.2 > 186.1 and 377.2 > 251.1 for IS, respectively. A simple gradient elution method was employed using an Ultimate XB-C_18_ column (2.1 mm × 50.0 mm, 5 μm; Welch, Shanghai, China). The mobile phase consisted of 10 mM of ammonium formate in water (phase A) and 0.2% formic acid in a mixture of methanol, acetonitrile, and isopropanol (75:15:15, v/v/v; phase B). The column oven and autosampler were set at 40 °C and 15 °C, respectively.

The total analysis time for each sample was 2.5 min, with a constant flow rate of 0.72 mL/min. The gradient elution program was as follows: 0–0.2 min: 20% B; 0.2–1.3 min: 20% to 88% B; 1.3–1.8 min: 88% B; 1.8–2.0 min: 88% to 20% B; 2.0–2.5 min: 20% B. This gradient elution program allowed for the effective separation and analysis of the compounds of interest.

The specific source and gas parameters used were as follows: collision gas set to medium, curtain gas maintained at 40 psi, ion source gas 1 set to 50 psi, ion source gas 2 adjusted to 55 psi, ion spray voltage set at 5,500 V, and ion spray temperature held at 600 °C. The transitions and optimized settings are listed in **Table 1**. Data acquisition and analysis were performed using the Analyst software package (Applied Biosystems, Version 1.6.3).

**Table 1.** Multiple reaction-monitoring parameters.

Compound	MRM transition (m/z)	Dwell time (ms)	DP (V)	EP (V)	CE (V)	CXP (V)
Baricitinib	372.3 > 186.1	70	64	10	48	10
	372.2 > 251.1	75	100	10	30	10
Baricitinib-d5	377.2 > 186.1	60	40	10	45	10
	377.2 > 251.1	60	40	10	39	10

CE, Collision Energy; DP, Declustering Potential; EP, Entrance Potential; CXP, Collision Cell Exit Potential

### 2.5. Method Validation

All method validation procedures adhered to bioanalytical method validation guidance provided by the US Food and Drug Administration (FDA) and the EMA [26,27].

#### 2.5.1. Selectivity, specificity, and carryover

Method selectivity and specificity were assessed by analyzing and comparing the peak areas of the analytes and IS in blank matrices, which included normal blank plasma or aqueous humor with the LLOQ samples. This analysis confirmed the absence of endogenous interference peaks. Six different sources of blank matrix were used to ensure the robustness of the data. Potential carryover effects were evaluated by comparing the signal intensities in double-blank samples after running the calibration standards at the upper limit of quantification (ULOQ; 200 ng/mL baricitinib) with the LLOQ samples five times. The impact of phospholipids in the matrix on the signal of baricitinib was evaluated by postcolumn infusion of the IS while an extracted blank sample or a solvent sample was being analyzed [28]. Notably, any chromatographic interference resulting from endogenous components should have a minimal impact on the accurate quantification of each compound. Baricitinib should not be eluted at a time when phospholipids are suppressing the signal of the IS. The peak area attributable to interfering components should not exceed 20% of the analyte response at the LLOQ and no more than 5% of the IS response in the LLOQ sample for each matrix. This criterion ensures reliable quantification of the target compounds in the analysis.

#### 2.5.2. Linearity

Linearity was assessed across a given concentration range for all analytes. Five calibration curves were constructed using eight freshly prepared calibration standards within the concentration range of 0.2–200 ng/mL over three consecutive days. Linear regression analysis was performed to fit the relationship between the peak area ratio (*y*) of baricitinib to IS and the nominal analytical concentration (*x*). A linear regression equation using a weighting scheme of 1/*x*^2^ was established. The calibration curves were considered acceptable if the accuracy of the back-calculated concentrations of the calibration standards was within ±20% for the LLOQ and within ±15% at all other levels, with at least three-quarters of the calibration points meeting this accuracy requirement.

#### 2.5.3. Precision and accuracy

Precision and accuracy assays were implemented for each QC sample, including LLOQ, LQC, MQC, and HQC. To evaluate method accuracy, we calculated the percentage ratio of each QC level to the nominal concentration as the mean of measured/nominal × 100%. Precision was evaluated by the coefficient of variation (CV), defined as the relative standard deviation (RSD) of each QC concentration level. Intra-run assays involved the analysis of six replicates at each QC concentration level in each analytical run. Inter-run assays were performed for each QC concentration level by pooling data from three analytical runs over three days. Accuracy (relative error) and precision (CV) below 15% (with the LLOQ threshold set at ≤20%) were deemed acceptable.

#### 2.5.4. Matrix effect, recovery, and dilution integrity

Extraction recovery was evaluated by comparing the QC samples (before the addition of the analyte and IS) with the samples that were spiked with analyte post extraction. Performance was expressed as the percentage of the peak areas of the analyte and IS in the preaddition QC sample versus the peak areas of the analyte and IS obtained from samples that were spiked with analyte post extraction. Extraction recovery was assessed at LQC, MQC, and HQC levels, with six parallel samples prepared for each.

Matrix effect assays were performed by analyzing six replicates of LQCs, MQCs, and HQCs, each prepared using blank plasma or aqueous humor from six different donors. Matrix effect assays in three replicates of lipemic or hemolyzed plasma were also performed. The matrix factor (MF) was determined by calculating the ratio of peak areas obtained from samples that were spiked with analyte post extraction to the peak areas obtained by spiking analyte into neat solutions. The IS-normalized MF (NMF) was calculated by dividing the MF of the analyte by that of the IS.

For both extraction recovery and NMF at each QC level, the accuracy should be within ±15% of the nominal concentration, and the precision (CV) should not be greater than 15%.

The dilution integrity was assessed using six determinations at concentrations exceeding the maximum calibration limit. The DQC samples were prepared and further diluted with blank plasma or aqueous humor at a ratio of one to four (v/v). The concentration of baricitinib in the matrix-spiked DQC samples was set to 80 ng/mL, which fell within the linear range. The calculated accuracy and precision did not exceed 15%.

#### 2.5.5. Stability

Analyte stability was evaluated in plasma using three levels of QC samples stored under four different storage conditions. Freeze-thaw stability was assessed by subjecting samples to three full freeze-thaw cycles (transitioning from –80 °C to 25 °C) at room temperature. Analyses were performed on QC samples stored at 25 °C for 24 h for short-term stability assessment. The long-term stability of the plasma samples was analyzed after simultaneous preservation at –20 °C and –80 °C for 14 and 45 days. QC samples were extracted at 4 °C and stored in an autosampler for 24 and 48 h to assess postprocess stability. Stability was determined by comparing the average analyte concentrations to the initial nominal concentrations, with stability defined as a percentage difference within ±15.0%.

### 2.6. Applicability of the Method for TDM

The utility of the validated method was assessed by analyzing plasma or aqueous humor samples from 10 patients with NIU. Inclusion criteria were as follows: (1) aged between 18 and 65 years; (2) met the diagnostic criteria for NIU, including intermediate uveitis, posterior uveitis, or panuveitis; (3) active uveitis at Day 1 and a baseline examination with at least one of the following: (a) active inflammatory chorioretinal lesions and/or inflammatory retinal vasculopathy; (b) ≥2+ anterior chamber cells (according to Standardization of Uveitis Nomenclature [SUN] criteria); (c) ≥2+ inflammatory vitreous opacity (according to SUN criteria). Patients with the following conditions were excluded: (1) infectious uveitis or uncontrolled tuberculosis or hepatitis B; (2) severe glaucoma or cataract unrelated to uveitis; (3) older adult or obese individuals or those with a history of deep vein thrombosis or pulmonary thrombosis, surgery or other conditions that led to their being bedridden, severe cardiovascular and cerebrovascular diseases, or uncontrolled systemic diseases such as hypertension and diabetes. All participants provided informed consent before inclusion in the study.

All patients were orally administered 4 mg of baricitinib (Olumiant^®^) once daily at 10:00 am. Whole blood was drawn into dipotassium ethylenediaminetetraacetic acid vacuum tubes immediately predose (0 h), and 0.5, 1, and 1.5 h post dose after administration of the second dose. Aqueous humor samples were drawn into sterile plastic tubes immediately before administration of the second dose (0 h). Whole blood was centrifuged at 1800 × g for 10 min, and the plasma and aqueous humor were stored at –70 °C until analysis. The trapezoid rule was used to calculate the area under the blood concentration–time curve over 1.5 h (AUC_0_–_1.5_), and then the median, first quartile, and third quartile of the AUC_0_–_1.5_ adjusted by the daily dose were calculated. Pearson correlation coefficients were used to assess the association between the concentration in aqueous humor and the concentration or AUC_0_–_1.5_ of plasma using R software (Version 4.2.1, http://www.r-project.org, accessed January 30, 2023). Statistical analysis and figure generation were performed using the tidyverse and ggplot2 packages.

## 3. Results and Discussion

### 3.1. Optimization of Chromatography and Mass Spectrometry

A 40 ng/mL solution of baricitinib and the IS was used to determine the mass spectrometric parameters. Consistent with previous reports [14], a more stable and higher signal-to-noise intensity ratio was observed in the ESI positive-ion mode. This generated the most abundant prominent protonated molecular ions [M + H]^+^ at 372 and 377 for baricitinib and its isotopic IS, respectively. Both analytes produced predominant fragment ions at m/z 186.1 and 251.1. Finally, we adopted 372.2 > 186.1 and 377.2 > 186.1 as the quantified mass transitions for baricitinib and IS, respectively, while the other two pairs of mass transitions were used for compound confirmation.

Because a single-step protein precipitation was sufficient to cover the sensitivity and concentration range, acetonitrile was chosen as the extracting solution due to its superior ability to yield cleaner and more efficient results than methanol. Following this step, the supernatants were reconstituted with an equivalent volume of 50% methanol containing 0.1% formic acid to further minimize the matrix effect and maximize ionization intensity.

To achieve optimal chromatographic separation, the composition of the mobile phase was systematically optimized. The aqueous phase consisted of various concentrations of ammonium formate or ammonium acetate solution, while the organic phase was evaluated in different stages. Initially, a binary mixture of methanol and acetonitrile was employed to balance the insufficient ionization efficiency associated with acetonitrile and the relatively high column pressure induced by methanol. Subsequently, the addition of a small proportion of isopropanol to this binary mixture improved peak shape by reducing tailing, yielding symmetric and sharp peaks. The final mobile phase comprised 10 mM of ammonium formate and 0.2% formic acid in water as the aqueous component and a ternary mixture of methanol, acetonitrile, and isopropanol (75:15:15, v/v/v) as the organic component.

Multiple commercially available C_18_ columns were evaluated to enhance chromatographic peak shape, peak width, and separation. The Ultimate XB-C_18_ column (2.1 mm × 50.0 mm, 5 μm) was selected due to its favorable peak shape, narrow peak width, and cost-effectiveness. Under the aforementioned chromatographic conditions, baricitinib and the IS were effectively separated with a retention time of 1.27 min within a total running time of 2.5 min, resulting in a shorter analysis time than prior methods [20–24].

### 3.2. Method Validation

#### 3.2.1. Selectivity, specificity, and carryover

**Figures 2** and **3** display the typical MRM chromatograms of baricitinib for the double-blank, LLOQ, and patient samples from plasma and aqueous humor. These chromatograms demonstrate the absence of any endogenous substances that interfere with baricitinib and the IS. The postcolumn infusion assay indicated that the IS was not coeluted with phospholipids from 1.0 to 1.3 min, demonstrating the negligible influence of phospholipids in both plasma and aqueous humor. Responses attributable to interfering components in the blank plasma and aqueous humor samples were less than 3.17% and 1.75% of the corresponding LLOQ samples, respectively. Both the analyte and the IS displayed peaks at 1.27 min.

**Figure 2.**
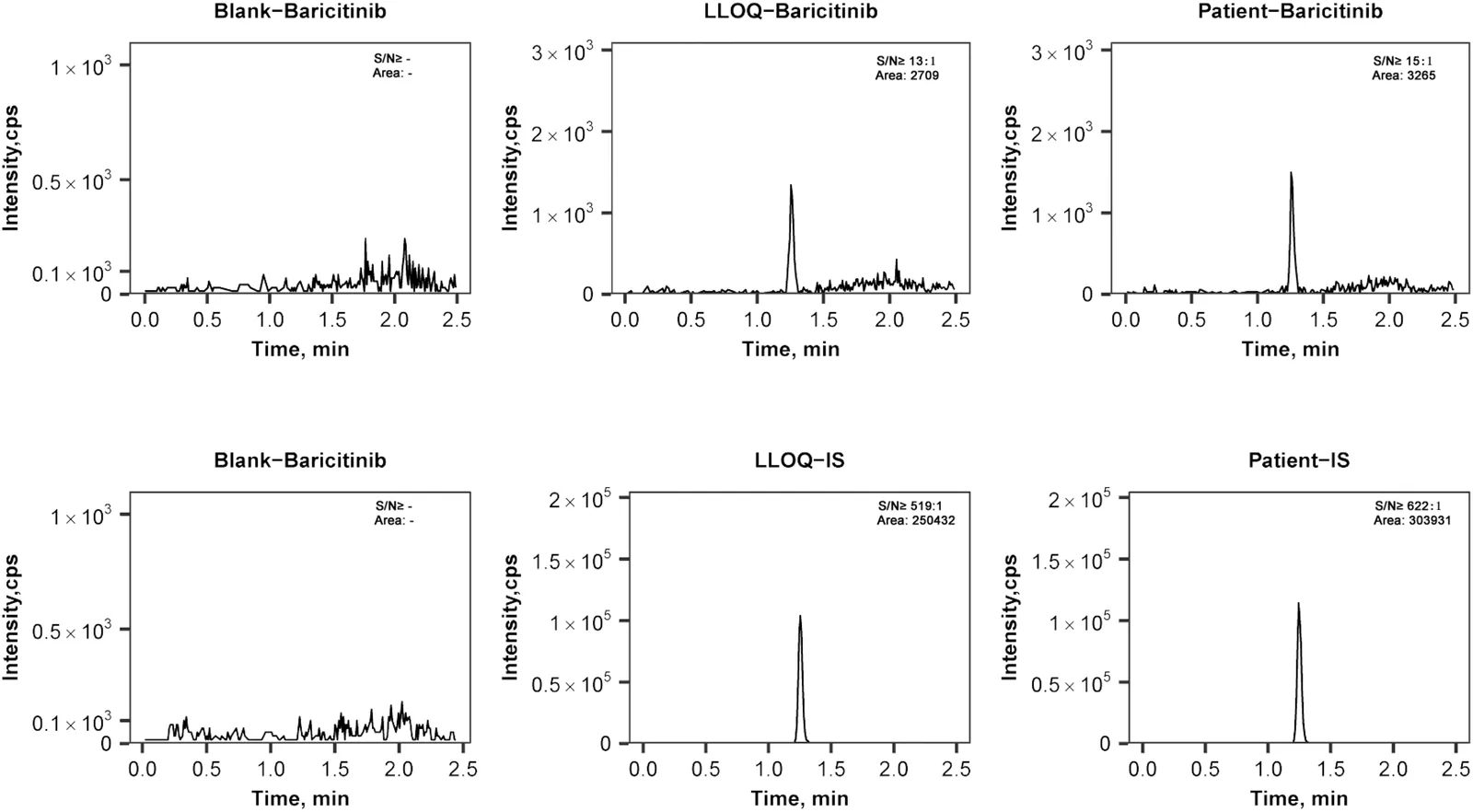
Typical MRM chromatograms of baricitinib and IS in double-blank plasma, LLOQ plasma sample, and plasma sample from patient (No. 4) collected immediately before (0 h) the second dosage administration.

**Figure 3.**
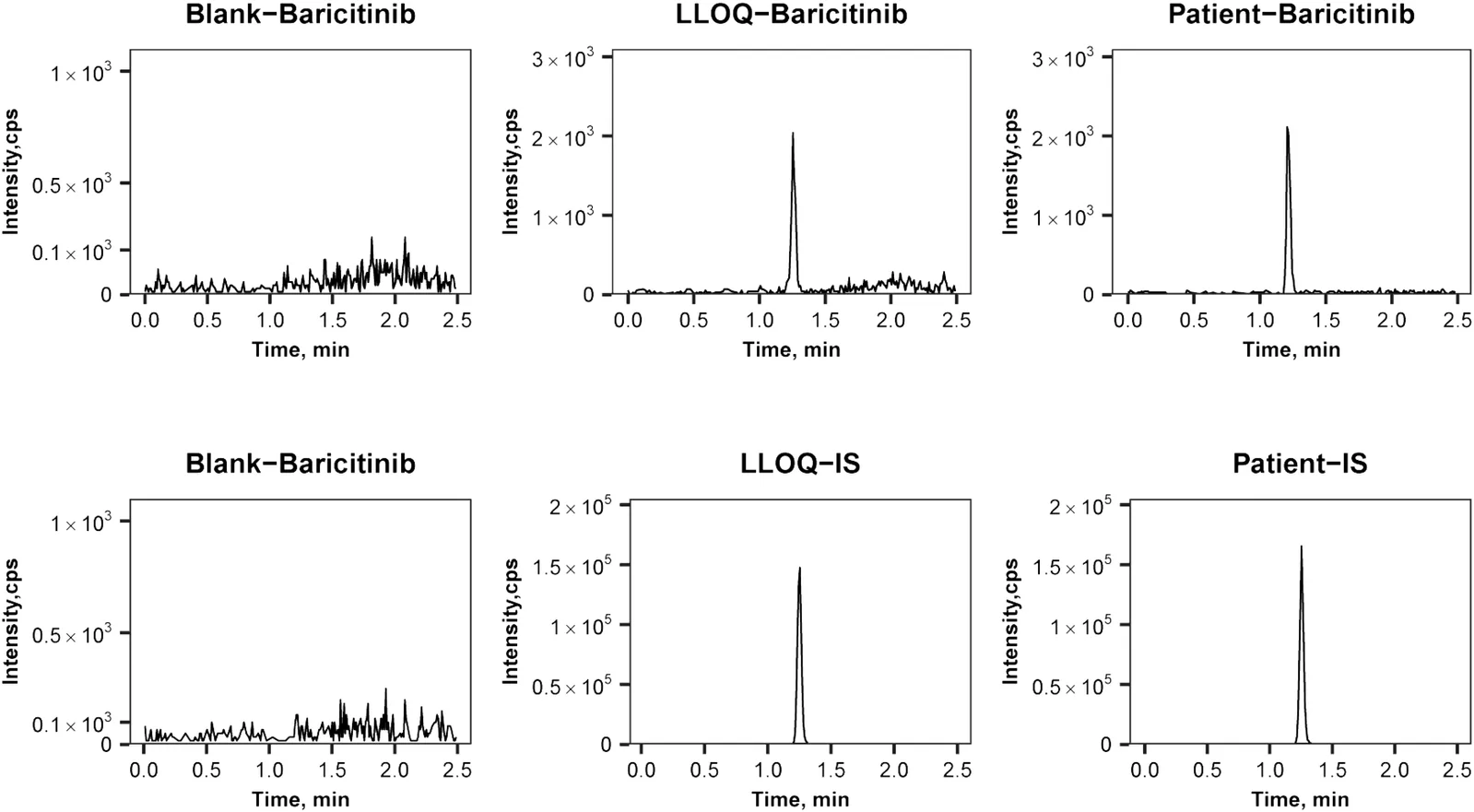
Typical MRM chromatograms of baricitinib and IS in double-blank aqueous humor, LLOQ aqueous humor sample, and aqueous humor sample from patient (No. 4) collected immediately before (0 h) the second dosage administration.

The carryover effect was minimal, as determined by comparing the peak areas of the double-blank samples injected after the ULOQ samples with those of the LLOQ samples. The average peak areas of the carryover blank samples were less than 0.46% and 0.33% of the LLOQ sample for baricitinib and IS in plasma, respectively, whereas the carryover effect was less than 0.06% and 0.69% for the same compounds in aqueous humor.

#### 3.2.2. Interference among analyte and IS

For the plasma matrix, the average peak areas of the double-blank and ULOQ samples without IS did not exceed 16% and 4% of the corresponding peak areas observed in the LLOQ samples, respectively. Similarly, these values did not exceed 14% and 3% for the aqueous humor matrix, respectively. This observation demonstrated excellent compliance with the acceptance criteria outlined by the FDA and EMA.

#### 3.2.3. Linearity, LLOQ, accuracy, and precision

The calibration curves exhibited linearity in both plasma and aqueous humor over a concentration range of 0.2–200 ng/mL, with determination coefficients (*r*^2^) higher than 0.99. The regression equations and *r*^2^ for these calibration curves are presented in **Table 2**, affirming the method’s satisfactory sensitivity and excellent linearity. Furthermore, the LLOQ of baricitinib was 0.2 ng/mL in both plasma and aqueous humor, ensuring a signal-to-noise ratio exceeding the baseline by 10-fold. This stringent criterion reinforces the reliability of concentration detection. The intra- and inter-run accuracy of baricitinib in plasma and aqueous humor ranged from 102.2% to 108.5% and 101.0% to 106.8%, respectively, while the intra- and inter-run precision ranged from 3.7% to 11.7% and 1.0% to 9.0%, respectively. All results indicated that the accuracy and precision of the validated method met the determination requirements.

**Table 2.** Intra- and inter-run accuracy and precision for the quantification of baricitinib in plasma or aqueous humor by LC-MS/MS (n = 6).

		Accuracy (%)	Precision (%)	Accuracy (%)	Precision (%)
Plasma	0.2	102.2 ± 11.9	11.7	102.7 ± 9.8	9.5
	0.6	107.7 ± 9.8	9.2	108.0 ± 7.1	6.6
	7.5	107.3 ± 3.1	2.9	108.5 ± 3.6	3.3
	150	104.9 ± 3.8	3.7	105.6 ± 3.6	3.4
Regression equation *y* = 0.05399*x* + 0.00201, *r*^2^ = 0.9995					
Aqueous humor	0.2	105.8 ± 9.0	8.5	106.8 ± 7.4	6.9
	0.6	103.0 ± 9.2	9.0	105.7 ± 8.0	7.6
	7.5	101.9 ± 1.0	1.0	103.2 ± 1.5	1.5
	150	104.2 ± 0.5	0.5	101.0 ± 4.1	4.1
Regression equation *y* = 0.0617*x* + 0.00151, *r*^2^ = 0.9989					

Accuracy presented as mean ± SD; precision presented as CV.

#### 3.2.4. Matrix effect, recovery, and dilution intensity

The results showed that the relative extraction recovery of baricitinib at all QC levels ranged from 86.5% to 94.5% in normal plasma and from 86.2% to 92.3% in aqueous humor, while the relative extraction recovery of the IS ranged from 85.3% to 99.9% in plasma and from 94.2% to 95.9% in aqueous humor (**Table 3**). The overall CV of the extraction recovery for all samples in both matrices did not exceed 15%, suggesting that the sample pretreatment method was reproducible and stable.

**Table 3.** Extraction recovery and matrix effect of baricitinib in plasma or aqueous humor.

Theoretical concentration (ng/mL)	Extraction recovery (%)		NMF (%)			
	Normal plasma	Aqueous humor	Normal plasma (n = 6)	Normal aqueous humor (n = 6)	Hemolyzed plasma (n = 3)	Lipemic plasma (n = 3)
0.6	88.3 ± 11.2	86.2 ± 2.5	107.4 ± 13.7	101.1 ± 5.9	105.6 ± 9.1	108.8 ± 12.7
7.5	94.5 ± 9.3	92.3 ± 4.4	101.2 ± 6.9	97.4 ± 6.6	103.7 ± 7.3	98.1 ± 7.8
150	86.5 ± 7.6	91.5 ± 0.5	98.3 ± 7.0	92.8 ± 0.9	109.6 ± 4.1	105.8 ± 9.4

Data presented as mean ± CV.

The NMF values of baricitinib varied from 98.3% to 107.4% in normal plasma, 92.8% to 101.1% in normal aqueous humor, 103.7% to 109.6% in hemolyzed plasma, and 98.1% to 108.8% in lipemic plasma. In addition, the CV of the NMF values in all matrices did not exceed 15% (**Table 3**). These results indicate that the use of acetonitrile as a deproteinization solvent, together with acetonitrile-water (50:50, v/v) dilution of sample supernatants, effectively removed matrix interference.

In terms of dilution integrity, the mean values of the calculated accuracy from six determinations diluted from the fivefold concentration of baricitinib with normal plasma or aqueous humor were 99% and 101.3%, respectively, with corresponding precision (CV) of 2.7% and 4.9%. Thus, the validated method was reliable and robust if the concentration range was within fivefold of the ULOQ.

#### 3.2.5. Stability

The stability results for baricitinib under various simulated conditions are presented in **Table 4**. The results indicated that baricitinib maintained reasonable stability when left at room temperature for 24 h, kept in the autosampler for 24 h, subjected to three freeze-thaw cycles, and stored at –80 °C for 320 days in normal plasma or aqueous humor. The degradation rate of baricitinib in whole blood maintained at 4 °C for 24 h was less than 4% with an RSD below 7.4%. Stock solution stability (last column of Table 4) reflects the stability of the baricitinib stock solution stored at –80 °C for 6 months, which showed accuracy of 85.3% to 105.4% and precision of 6.4% to 9.3%. These findings indicate that this method is stable for processing plasma or aqueous humor samples over an extended period.

**Table 4.** Stability of baricitinib in plasma or aqueous humor under different conditions (n = 3).

Theoretical concentration (ng/mL)		Accuracy (%)					
		Blood at 4 °C for 24 h	At room temperature for 24 h	In autosampler for 24 h	Three Freeze-thaw cycles	–80 °C for 320 days	Stock sol. stability at –80 °C for 6 months
Plasma	0.6	106.0 ± 7.8	109.3 ± 4.5	103.5 ± 2.9	109.7 ± 10.0	93.9 ± 4.5	91.3 ± 6.0
	150	106.7 ± 1.6	102.6 ± 2.4	107.8 ± 2.8	111.1 ± 2.4	110.0 ± 2.1	96.4 ± 9.0
Aqueous humor	0.6	-	98.3 ± 4.4	106.1 ± 2.3	102.4 ± 2.4	99.1 ± 5.6	-
	150	-	101.6 ± 5.7	103.2 ± 3.0	103.5 ± 3.8	103.2 ± 3.0	-

Data presented as mean ± CV.

### 3.3. Clinical Application

The plasma concentration–time profiles are shown in **Figure 4**, and the demographic and laboratory characteristics are summarized in **Table 5**. The plasma concentrations of baricitinib at 0 h (before the second dose) and at 0.5, 1, and 1.5 h after the second dose were 2.25 (1.79, 3.31), 30.18 (11.27, 44.02), 44.64 (30.46, 60.02), and 43.53 (36.87, 54.34) ng/mL, with CV values of 49.6%, 81.0%, 48.6%, and 30.4%, respectively. The calculated AUC_0_–_1.5_ and daily dose-normalized AUC_0_–_1.5_ (AUC_0__–__1.5_/D) were 53.30 (40.62, 87.0) and 13.30 (10.15, 18.57) ng·h/mL/mg, respectively, with both CV values at 51.4%. Furthermore, half of the patients did not exhibit a delayed absorption peak within 1.5 h, indicating that not all patients achieved the peak concentration within 1.0 h. This finding is inconsistent with results from earlier phase 1 and 2 clinical trials [17]. One plausible explanation for this discrepancy is that our study, conducted in a real-world clinical setting, did not strictly require patients to take the medication on an empty stomach, and some may have taken it after a meal, despite the fact that oral bioavailability is not affected by food.

**Figure 4.**
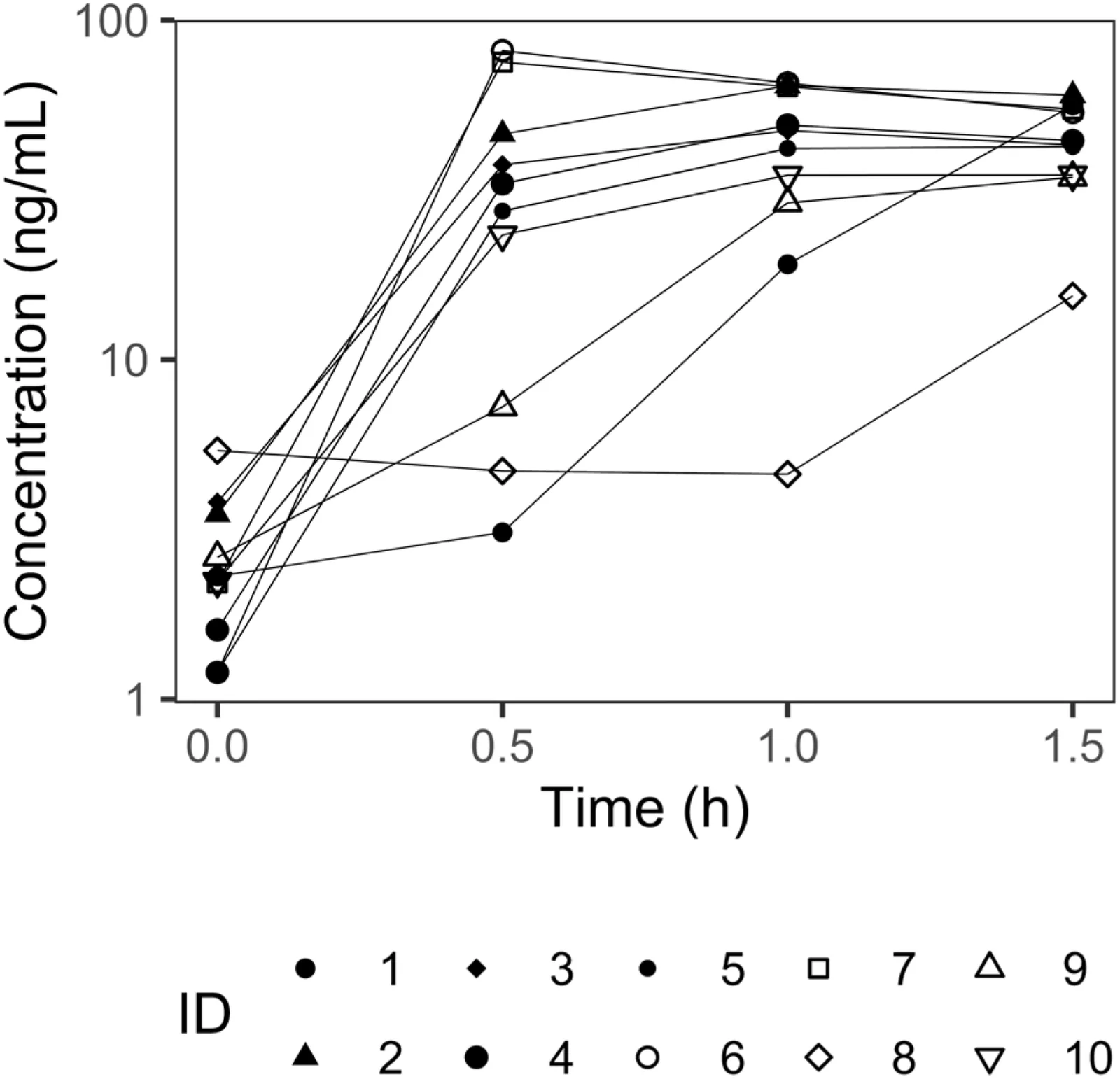
Plasma concentration versus time profiles in 10 patients with NIU after the second dose (baricitinib tablet, 4 mg once daily).

**Table 5.** Patient demographics and disease characteristics.

Characteristic	N (%) or median (Q1, Q3)
Sex (male/female)	4/6
Age (years)	38.5 (32.7, 44.2)
Body weight (kg)	64.5 (60.2, 71.8)
Type of uveitis	
Anterior uveitis	3 (30)
Posterior and/or panuveitis (and/or intermediate)	7 (70)
Unilateral	2 (20)
Bilateral	8 (80)
Etiology	
Vogt-Koyanagi-Harada syndrome	7 (70)
Behçet’s syndrome	1 (10)
Others	2 (20)
Comorbidities/complications	
Glaucoma	1 (10)
Ocular hypertension	2 (20)
Epiretinal membrane	7 (70)
Cataract	3 (30)
Retinal ischemia within the macula	3 (30)
Retinal ischemia within peripheral retina	3 (30)
Alanine aminotransferase (IU/L)	17.0 (10.2, 25.5)
Aspartate aminotransferase (IU/L)	16.0 (13.2, 29.5)
Estimated glomerular filtration rate^a^ (L/h)	105 (102.3, 116.8)
Concomitant medication	
Methotrexate	2 (20)
Methotrexate dose (mg/week)^b^	17.5 ± 3.5

a Calculated according to the Chronic Kidney Disease Epidemiology Collaboration equation and converted to liters per hour.

b Represented as mean ± SD.

The trough concentration (C_min_) of baricitinib after the first dose in the anterior chamber was 0.94 (0.89, 1.16) ng/mL, with a CV of 20.2%, which was significantly lower than the plasma C_min_ (49.6%). Furthermore, significant correlations between the concentrations in the aqueous humor at 0 h and the plasma concentrations at 0, 0.5, 1, and 1.5 h, as well as the corresponding plasma AUC_0_–_1.5_, were not observed (**Figure 5**). These findings indicate that drug concentrations in the anterior chamber are less susceptible to fluctuations in plasma concentrations and that it may not be feasible to predict the therapeutic efficacy of ocular diseases by monitoring drug concentrations in plasma.

**Figure 5.**
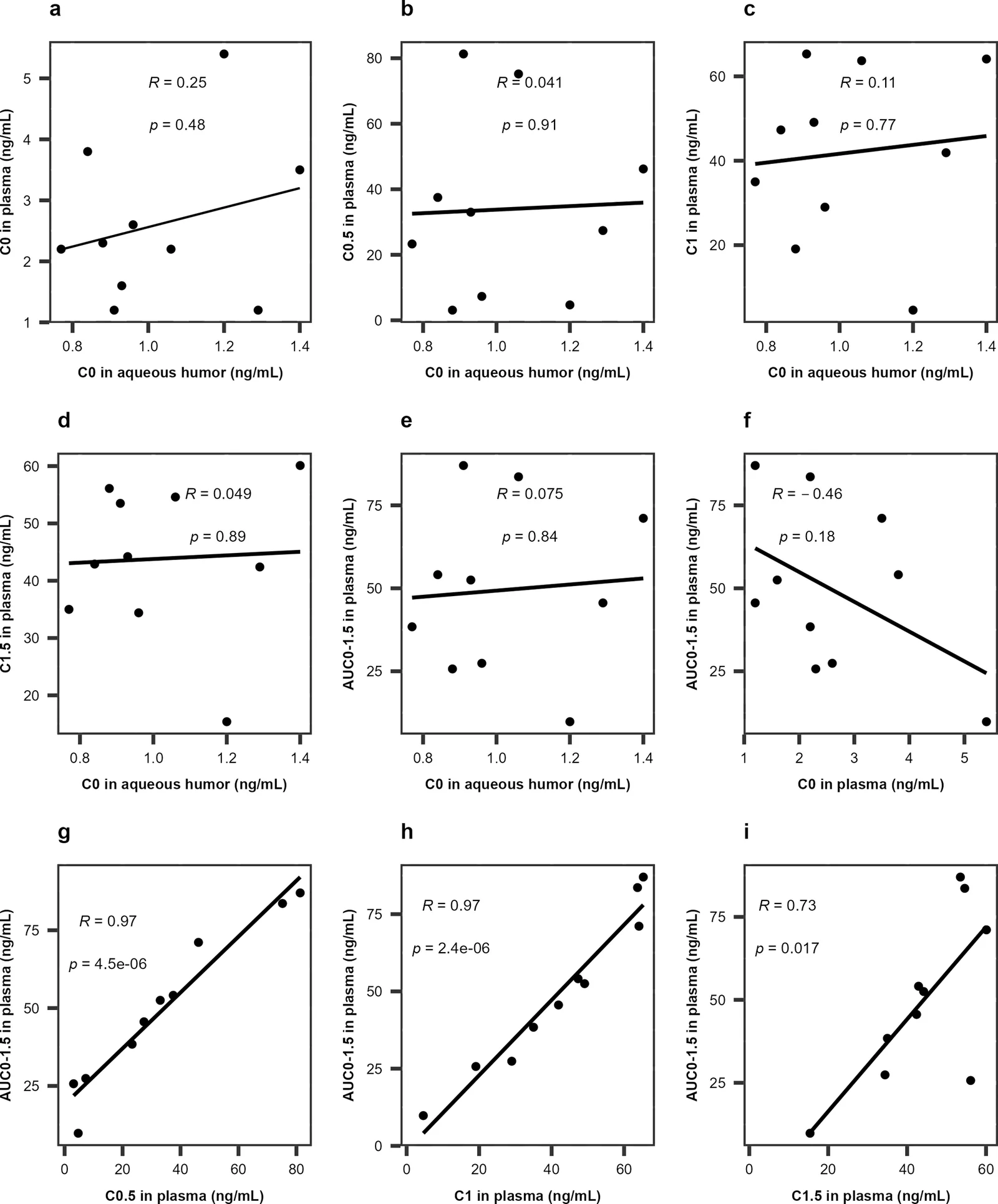
Pearson’s correlation between aqueous humor and plasma. **a–d**, BARCI levels in plasma at 0 h (C_0_), 0.5 h (C_0.5_), 1 h (C_1_), and 1.5 h (C_1.5_) versus BARCI levels in aqueous humor at 0 h. **e**, BARCI AUC_0–1.5_ in plasma versus BARCI levels in aqueous humor at 0 h. **f**, **f–i**, plasma BARCI AUC_0–1.5_ versus plasma BARCI concentrations at 0, 0.5, 1, and 1.5 h.

### 3.4. Comparison with Previous Studies

Several methods have been established to quantify baricitinib in the plasma of rats and humans [20–24]. To date, these quantification methods have been validated by LC-MS/MS in MRM-positive mode, with three reports using isotopic IS and analysis times exceeding 2.8 min [23–25]. In addition, the application of microsampling technology in one PK conducted in pediatrics [23], although innovative, presented high technical barriers with no superior cost-effectiveness. The latest published method by Cafaro et al. [25], which is similar to ours, requires only a small sample volume, a straightforward sample preparation protocol, and a low detection limit. However, their method did not cover both peak and trough points to verify its applicability, nor did it explore drug concentrations in aqueous humor. Furthermore, all other studies based on human plasma used liquid-liquid extraction for sample pretreatment, which is complicated and time-consuming. In summary, no reports on the determination of baricitinib in aqueous humor are currently extant. This study provides improvements by using LC-MS/MS; for instance, the sensitivity was adequate without the need for complicated and time-consuming preprocessing, the analytical time was shorter, and the matrix effect was eliminated.

An important factor contributing to the success of ocular therapy is the ability of drug molecules to penetrate targeted ocular tissues at effective concentrations. Moreover, aqueous humor is an accessible site for the systemic administration of baricitinib. Given the presence of the blood-ocular barrier, baricitinib in the aqueous humor is closer to the ciliary body or choroid than that in plasma, and monitoring its PK in the aqueous humor is relatively more suitable than plasma for evaluating the exposure-efficacy relationship in patients with NIU. Nevertheless, it must be clearly recognized that aqueous humor remains a surrogate matrix. Researchers have advocated the development of baricitinib ophthalmic liposomes, as direct ocular administration may achieve better penetration into target tissues than oral administration, potentially enhancing therapeutic efficacy [29].

This study also presents some limitations. First, plasma concentrations at the terminal elimination phase were not monitored during the second dosage interval. Therefore, AUC_0–1.5_ could not fully substitute for the AUC_24_ of baricitinib in plasma. Second, the small sample size in our study may limit the generalizability of the PK variability observed. In addition, variability in drug administration (fasted vs. fed states) may have contributed to the observed PK variability but was not controlled in this real-world study. Last, the use of Pearson correlation provides only a preliminary assessment of the relationship between plasma and aqueous humor concentrations. More robust analyses (e.g., population PKs) would better characterize the compartmental dynamics and exposure-effect relationship in NIU cohorts but would require larger sample sizes. These limitations highlight opportunities for future research to optimize baricitinib TDM in ocular diseases.

## 4. Conclusion

This method provides a reliable and valuable reference for promoting future clinical studies and improving the therapeutic management of baricitinib in patients with NIU. The implementation of population PK/pharmacodynamic studies is urgently needed to establish the exposure-efficacy relationship of baricitinib in NIU cohorts.

## Data Availability

The data generated and analyzed in this study are available on request from the corresponding author.

## Abbreviations

AUC: Area under the curve
BARCI: Baricitinib
CV: Coefficient of variation
DMSO: Dimethyl sulfoxide
DQC: Dilution quality control
EMA: European Medicines Agency
FDA: US Food and Drug Administration
HQC: High-quality control
IMID: Immune-mediated inflammatory disease
IU/L: International units per liter
IS: Internal standard
JAK: Janus kinase
JAK-STAT: Janus kinase signal transducer and activator of transcription
LLOQ: Lower limit of quantification
LQC: Low-quality control
MF: Matrix factor
MQC: Medium-quality control
NIU: Noninfectious uveitis
NMF: Internal standard–normalized matrix factor
PK: Pharmacokinetic
QC: Quality control
RSD: Relative standard deviation
SUN: Standardization of Uveitis Nomenclature
TDM: Therapeutic drug monitoring
ULOQ: Upper limit of quantification
v/v: Volume/volume percentage

## Credit Author Statement

All authors have read and agreed to the published version of the manuscript.
